# Implementing the battery-operated hand-held fan as an evidence-based, non-pharmacological intervention for chronic breathlessness in patients with chronic obstructive pulmonary disease (COPD): a qualitative study of the views of specialist respiratory clinicians

**DOI:** 10.1186/s12890-022-01925-z

**Published:** 2022-04-06

**Authors:** Tim Luckett, Mary Roberts, Tracy Smith, Maja Garcia, Sarah Dunn, Flavia Swan, Caleb Ferguson, Slavica Kochovska, Jane L. Phillips, Mark Pearson, David C. Currow, Miriam J. Johnson

**Affiliations:** 1 IMPACCT (Improving Palliative, Aged and Chronic Care Through Clinical Research and Translation), Faculty of Health, University of Technology Sydney (UTS), Building 10, Level 3, 235 Jones St, Ultimo, Sydney, NSW 2007 Australia; 2 Department of Respiratory and Sleep Medicine, Westmead Hospital, Sydney, NSW Australia; 3 Ludwig Engel Centre for Respiratory Research, Westmead Institute for Medical Research, Sydney, NSW Australia; 4 The University of Sydney at Westmead Hospital, Sydney, NSW Australia; 5 Respiratory Medicine Clinic, Prince of Wales Hospital, Sydney, NSW Australia; 6 Wolfson Palliative Care Research Centre, Hull York Medical School, University of Hull, Kingston Upon Hull, Yorkshire UK; 7 Western Sydney Nursing & Midwifery Research Centre, Blacktown Clinical & Research School, Western Sydney Local Health District, Western Sydney University, Blacktown Hospital, Sydney, NSW Australia; 8 School of Nursing, Queensland University of Technology, Brisbane, QLD Australia

**Keywords:** Breathlessness, COPD, Non-pharmacological management, Qualitative

## Abstract

**Introduction:**

The battery-operated hand-held fan (‘fan’) is an inexpensive and portable non-pharmacological intervention for chronic breathlessness. Evidence from randomised controlled trials suggests the fan reduces breathlessness intensity and improves physical activity in patients with a range of advanced chronic conditions. Qualitative data from these trials suggests the fan may also reduce anxiety and improve daily functioning for many patients. This study aimed to explore barriers and facilitators to the fan’s implementation in specialist respiratory care as a non-pharmacological intervention for chronic breathlessness in patients with chronic obstructive pulmonary disease (COPD).

**Methods:**

A qualitative approach was taken, using focus groups. Participants were clinicians from any discipline working in specialist respiratory care at two hospitals. Questions asked about current fan-related practice and perceptions regarding benefits, harms and mechanisms, and factors influencing its implementation. Analysis used a mixed inductive/deductive approach.

**Results:**

Forty-nine participants from nursing (n = 30), medical (n = 13) and allied health (n = 6) disciplines participated across 9 focus groups. The most influential facilitator was a belief that the fan’s benefits outweighed disadvantages. Clinicians’ beliefs about the fan’s mechanisms determined which patient sub-groups they targeted, for example anxious or palliative/end-stage patients. Barriers to implementation included a lack of clarity about whose role it was to implement the fan, what advice to provide patients, and limited access to fans in hospitals. Few clinicians implemented the fan for acute-on-chronic breathlessness or in combination with other interventions.

**Conclusion:**

Implementation of the fan in specialist respiratory care may require service- and clinician-level interventions to ensure it is routinely recommended as a first-line intervention for chronic breathlessness in patients for whom this symptom is of concern, regardless of COPD stage.

## Introduction

Chronic breathlessness is a common and burdensome symptom across a range of chronic illnesses, including cancer, heart failure and respiratory diseases including chronic obstructive pulmonary disease (COPD). It is defined as “breathlessness that persists despite optimal treatment of underlying pathophysiology and that results in disability” ([1], page 1).

While chronic breathlessness cannot be cured, it can be managed with non-pharmacological and pharmacological interventions aimed at modulating the person’s perception of breathlessness and response to it [2]. One such intervention is the battery-operated hand-held fan (‘fan’). Four meta-analyses, conducted across chronic diseases [3, 4] and in advanced cancer more specifically [5, 6], have suggested that the fan decreases breathlessness intensity. In a pooled analysis of two trials, it was also found that more than half of patients reported an increase in physical activity [7]. Moreover, qualitative sub-studies from three trials suggest that over 80% of participants perceived benefits in the broadest sense, including not only improvements in the sensory (intensity) dimension of breathlessness, but also benefits in affective (reduced panic) and impact (faster recovery after activity) dimensions, as well as increased confidence in engaging with activities of daily living [8].

The fan is recommended for breathlessness by international clinical practice guidelines not only for everyday breathlessness but also for managing acute-on-chronic episodes or ‘crises’ [9]. Acute-on-chronic breathlessness often leads to unplanned Emergency Department visits and hospitalisations [10], a substantial proportion of which might be prevented if patients had better training in breathlessness self-management [11, 12]. While no direct evidence is available for the fan’s potential to reduce avoidable Emergency presentations, randomised controlled trial (RCT) evidence that it can improve recovery time [13] alongside qualitative evidence summarized above for reductions in panic might plausibly help patients regain feelings of control that patients have reported are key to whether or not they seek Emergency assistance [14]. Given the fan is inexpensive and readily available for patients to purchase outside the health system, any potential to reduce preventable hospital use would make it highly cost-effective.

Previous research has also identified no confirmed risk of harm and few disadvantages from using the fan. Recent guidance from Public Health Ontario (2021) concluded that concerns about fans of any kind spreading COVID-19 and other infections remain “*theoretical*”, though caution has been recommended in long-term care facilities for the elderly [15]. Other disadvantages are limited to perception of stigma and discomfort in cooler weather reported by a minority of patients [8]. Not only is the fan safer than many medications but some patients report that it decreases their reliance on beta-agonist metered dose inhalers, potentially reducing unwanted side-effects [16].

The fan’s potential for benefit, few disadvantages and low cost combine with its portability to warrant its implementation as a ‘first-line’ intervention for amelioration of chronic breathlessness that should be recommended to all affected patients alongside disease-directed treatments [9, 17, 18]. However-as for many evidence-based interventions-specific efforts may be needed to drive implementation. Some patients will learn about the fan from consumer-facing organizations for chronic lung disease, which promote the fan alongside other non-pharmacological interventions through their websites and other patient resources [19, 20]. However, patients who seek out information from this source are likely to be already ‘activated’ in self-management [21], while patients with lower self-efficacy may instead rely on healthcare encounters to encourage health-related behaviours. One such opportunity is presented by “breathlessness services”, a relatively new model of care specifically designed to train patients in self-management using non-pharmacological interventions [22]. However, few breathlessness services are available worldwide, making specialist respiratory care the most common point of contact at which to implement the fan for patients with chronic lung disease. Unfortunately, however, a study conducted at one Australian breathlessness service found that the majority of COPD patients referred had never heard of the fan previously, despite receiving previous outpatient and inpatient specialist respiratory care and pulmonary rehabilitation [23].

To date, no research has explored clinician barriers and facilitators to implementing the fan for chronic breathlessness in specialist respiratory care [24]. However, research on non-pharmacological interventions more generally suggests that barriers occur mostly at the clinician level, rather than at the level of the patient or health service [25]. Clinicians may focus on biomedical management of disease rather than a biopsychosocial approach that prioritises optimising quality of life [26], lack awareness of the evidence surrounding non-pharmacological management of breathlessness, and/or have a mistaken belief that ‘nothing more can be done’ once the underlying disease has been optimally treated [25, 27].

In addition to the above barriers that are common across non-pharmacological interventions, the fan might face unique challenges to implementation. Patients in qualitative studies have voiced surprise that such an inexpensive, everyday item can be effective where other interventions have failed [8], and it seems likely that clinicians may be similarly dismissive unless they are familiar with evidence for the fan’s efficacy. Further, the fan has a number of physiological and psychological mechanisms that are still poorly understood [28, 29], potentially confusing clinicians regarding the pathway to benefit. Further research is needed to explore whether these and other barriers are impeding implementation of the fan in specialist respiratory care and what opportunities there might be for driving translation into practice.

The aim of the current study was to explore barriers and facilitators to implementation of the fan in specialist respiratory care as a non-pharmacological management intervention for chronic breathlessness in patients with COPD.

### Design and methods

The study used a qualitative method with pragmatic orientation to explore clinicians’ perceptions of barriers and facilitators to implementation of the fan in specialist respiratory care. We focused on clinicians’ perceptions because of evidence above that barriers to non-pharmacological management of breathlessness tend to stem from clinician knowledge, attitudes and beliefs, and to enable us to explore appearance and mechanism-related issues unique to the fan.

The study took place between December 2020 and April 2021 and was approved by South Eastern Sydney Local Health District Human Research Ethics Committee (2020/ETH02615). All participants gave informed consent to participate.

The study has been reported in accordance with the Consolidated Criteria for Reporting Qualitative Research (COREQ) [30].

### Participants

Participants were clinicians of any discipline (medical, nursing or allied health) working in specialist respiratory care at either of two quaternary referral and teaching hospitals in Sydney, Australia. Clinicians could be working in outpatient, inpatient or pulmonary rehabilitation services.

Rather than aim for a target sample size, the opportunity to participate was extended to all clinicians working in specialist respiratory care at the two hospitals. Participants were approached by a clinical investigator at each hospital via electronic mailing lists and announcements at clinician meetings. To minimize selection bias and ensure inclusion of a range of disciplinary and fan-related perspectives, data collection took place at the end of regular clinical meetings and training sessions. Clinicians who did not wish to participate were given the opportunity to leave before focus groups commenced.

### Data collection

Focus groups were used in preference to one-to-one interviews because they offer the most efficient method for enabling individual perspectives to be compared and integrated to identify group norms and points of difference, providing the topic is narrowly focused and not private [31, 32]. Focus groups were carried out face-to-face at each hospital where possible or using video conference when COVID-19 physical distancing restrictions were in place. Focus groups were facilitated by a male academic and social scientist with no previous relationship with participants (TL), with notes being taken by a female nurse and/or doctor who worked as part of the teams (MR, SD or TS). All these team members had prior experience of facilitating focus groups and conducting qualitative analysis, and a research interest in non-pharmacological management of chronic breathlessness.

At the beginning of focus groups, participants were asked to complete a brief proforma on their clinical experience and the proportion of patients with COPD and chronic breathlessness to whom they currently recommended the fan (‘all or nearly all’, ‘most’, ‘some’, ‘none’). This information was submitted to the team anonymously either in paper form (face-to-face) or electronically (video-conferencing), and was not available to facilitators during the focus group.

To minimise social desirability bias [33], the focus group preamble explicitly invited a range of perspectives on the fan, acknowledging the absence of previous research on clinician perceptions and practice. Questions started by inviting anyone who did not recommend the fan to discuss their reasons. Questions were then opened to all participants, and focused on perceived benefits and problems and any other factors influencing whether they recommended the fan. Participants who indicated during the discussion that they recommended the fan to some but not all patients were asked how they selected. Participants who had recommended the fan to any proportion of patients were asked how confident they felt in supporting patients to use it for optimal benefit, and to recall the factors that had first prompted them to implement the fan in their practice. Participants were also invited to comment on the fan’s appearance and mechanism.

Focus groups were audio-recorded and transcribed verbatim, with transcripts imported to NVivo version 12 (QSR) for management. Transcripts were not returned to participants for comment or correction.

### Analysis

Analysis used an integrated approach specifically designed for developing healthcare interventions that includes both inductive and deductive components to ensure that results built on previous understanding of clinician behaviour as well as remained open to unexpected insights [34]. Analysis commenced with line-by-line inductive coding, before using an established theoretical framework to transition to interpretative themes. The Integrated Behavioural Model (IBM) [35] was chosen because it: provided useful insights in similar qualitative studies exploring health professionals’ knowledge, beliefs and attitudes [36–38]; enabled a nuanced exploration of clinicians’ ‘intention to act’, which we anticipated to be the most influential factor within a clinical decision-making context; and considered factors beyond intention such as ‘environmental constraints’. The IBM posits that intention to act is primarily determined by a person’s attitudes, perceptions regarding other people’s attitudes and behavior (i.e. norms), and perceived control over the behaviour. Additional themes were developed inductively where they proved a poor fit for constructs in the IBM. Analysis was conducted by a medical student with experience in similar analysis (MG), with iterative discussion of the line-by-line coding structure with TL and interpretative themes with nursing and medical members of the investigator team (MR and TS).

## Results

A total of 9 focus groups were conducted, 4 at hospital #1 and 5 at hospital #2. Forty-nine clinicians participated in total (22 at hospital #1 and 27 at hospital #2), with numbers in each focus group ranging between 4 and 7. Samples from both hospitals included nurses, doctors and allied health professionals. Participants at hospital #1 were generally more likely to recommend the fan to ‘all/nearly all’ patients than those at hospital #2 (8/22 versus 2/27 respectively), but the proportions recommending to ‘most’ patients were similar (7/22 and 10/27 respectively). Focus groups lasted between 19 and 37 min. Further participant characteristics are summarized in Table 1.

**Table 1:** Characteristics of 49 clinicians from specialist respiratory care who participated in 9 focus groups exploring barriers and facilitators to implementing the battery-operated hand-held fan for chronic breathlessness in patients with COPD

Characteristic	N = 49 (%)
Gender	
Female	36 (73.5)
Male	13 (26.5)
Setting	
Inpatient	34 (69.4)
Inpatient/outpatient	8 (16.3)
Discipline	
* Medical*	*13 (26.5)*
Advanced trainee	12 (24.5)
Consultant	1 (2)
* Nursing*	*30 (61.2)*
CNC	1 (2)
CNE	1 (2)
CNS	2 (4.1)
EEN	3 (6.1)
RN	21 (42.9)
* Allied health*	*6 (12.2)*
Physiotherapist	5 (10.2)
OT	1
Years working in respiratory care	
< 1 year	6 (12.2)
1–5 years	23 (46.9)
6–10 years	8 (16.3)
> 10 years	12 (24.5)
“To what proportion of patients with COPD and chronic breathlessness do you currently recommend the hand-held fan?”	
All or nearly all	10 (20.4)
Most	17 (34.7)
Some	19 (38.8)
None	3 (6.1)

The italics represent totals for the section below them - for example medical is comprised of bot advanced trainees and consultants

CNC, clinical nurse consultant; CNE, clinical nurse educator; CNS, clinical nurse specialist; EEN, endorsed enrolled nurse; RN, registered nurse; OT, occupational therapist

Nearly all line-by-line codes could be mapped to the IBM framework. Findings relating to each of the framework’s constructs are summarized in Table 2 and described in more detail as follows.

**Table 2:** Barriers and facilitators to implementing the hand-held fan for breathlessness in specialist respiratory care as identified by clinician focus groups and classified using the Integrated Behavioural Model (IBM) [35]

IBM construct		Barriers	Facilitators
Instrumental attitude	Knowledge and beliefs about fan-related benefits and harms	Most participants lacked a knowledge of the fan’s evidence-base A small number of participants were concerned that patients might become over-reliant on the fan	Clinician awareness of evidence for the fan might benefit from greater inclusion in clinical practice guidelines None of the participants believed the fan posed a serious risk of harm so were more prepared to ‘try it’ even if unaware of evidence for benefit
	Knowledge and beliefs about the fan’s mechanism	Participants who believed the fan’s mechanism to be mostly psychological recommended it only to anxious patients Most participants reserved the fan for ‘end stage’ disease None of the participants considered the fan suitable during an exacerbation	Educational efforts should focus on persuading clinicians to recommend the fan to any patient with COPD for whom breathlessness impacts on daily life, regardless of anxiety and disease stage Use of the fan during exacerbations should be promoted in line with ATS guidance for breathlessness ‘crises’ [9]
Experiential attitude		Participants were much more motivated by the fan’s benefit/harm profile than whether it appeared ‘cheap’ or ‘plasticky’	None relevant
Normative beliefs	Descriptive norms	Participants’ views were relatively unaffected by colleagues, being much more influenced by patient reports regarding efficacy	Patient advocacy has been a key facilitator to date Clinical champions may have potential but are as yet untested
	Subjective norms	At a setting level, participants tended to consider outpatient and community services more appropriately placed to implement the fan than inpatients, where patients were acutely unwell At a specialty level, some participants expected specialist palliative care to recommend the fan rather than respiratory care At a discipline level, some nurses and allied health professionals considered doctors to be more focused on pharmacological than non-pharmacological management, but doctors refuted this At a clinician level, participants agreed that anyone could recommend the fan but were unclear whose role this should be	Allocate the fan within the scope of practice for respiratory care within each setting and discipline for all or selected roles
Personal agency	Perceived behavioural control	Participants expressed a high level of autonomy with regard to the fan, rendering this no barrier to implementation	None relevant
	Self-efficacy	Most participants were uncertain which type of fan to recommend and how to train patients to use it optimally	Promote types of fan supported by emerging evidence [59] Further research is required to identify the optimal approach to training patients in using the fan
Environmental factors		A lack of hand-held fans in the inpatient setting posed a significant barrier	Establish locally-contextualised models for ensuring fans are available to patients in the inpatient setting
Salience and habit		In a time-pressured procedurally-driven inpatient setting, implementation was perceived to be hindered by the fan not being part of a standard protocol	Include the fan in ward protocols

ATS, American Thoracic Society

### Attitudes

#### Instrumental attitude

##### Knowledge and beliefs about fan-related benefits and harms

The most influential factor determining clinicians’ willingness to recommend the fan appeared to be their ‘instrumental attitude’—in particular, the degree to which they believed that patient benefit outweighed any disadvantages or harms.

When asked about barriers to implementing the fan, some participants highlighted a “lack of knowledge or a lack of awareness of how a fan can help” (Hospital 1, Pulmonary rehabilitation), with doctors indicating they hadn’t learned about fans in medical school or post-graduate training. Only a few participants appeared familiar with research evidence for the effectiveness of the fan, which they had heard about through related training. Even these participants identified clinical experience as a more important driver.

“She [a trainer] told us about some research that had been done in England about the fan how it can help people with their breathlessness. So, I read up about it, started recommending to patients, and found that it does really help them with their breathlessness.” Hospital 1, Nurse

Most participants reported learning about the fan through patients’ endorsement, and perceived that patients usually learned through the same source.

“Patients who are already using the fan encourage other people to use the fan.” Hospital 1, Nurse

Participants perceived patients to have reaped a range of benefits from using the fan. For some, this included a patient perception that the fan had relieved breathlessness intensity.

“My patients that I've had, they've reported that they feel a lot less breathless with the fan on and they tend to self-initiate the hand held fan when they do start feeling breathless, as well.” Hospital 1, Advanced trainee

Participants in four focus groups also reported observing psychological benefits from the fan, including reductions in anxiety and feelings of panic.

“Yeah, and in saying that, I've had patients say it eases anxiety when they're really short of breath; rave about it.” Hospital 1, Nurse

The fan was also perceived to increase patients’ confidence, allowing them to extend their activities of daily living.

“The patients have said that it makes them feel less breathless and more confident in managing attacks of breathlessness. So they feel more confident leaving the house, for example. Yeah. That's probably the main things they've said.” Hospital 1, Advanced trainee

One participant also described the benefit of patients using the fan during exercise.

“I also found that during the exercise program, patients cope better doing their exercises if they are holding a fan while they're exercising.” Hospital 1, Nurse

None of the participants considered there to be serious harms associated with using the fan. Potential harms that were raised by participants but dismissed as minimal included risk of injury from the blades (*“that stuff [the blades are made of] is soft” Hospital 1, Advanced trainee)* and fire from batteries (“*lithium batteries burn hot and durably, I think*” *Hospital 2, Physician*). The lack of perceived harms encouraged participants to take a “*why not try it?*” (*Hospital 1, Nurse*) approach to recommending the fan, even when they were uncertain whether a given patient might benefit.

“If I was worried that it would do the patient harm, yes, I would have more questions and reservations about it, but I just don't feel that way about hand held fans.” Hospital 1, Advanced trainee

Participants were more divided on the risk of transmitting COVID-19, with those at hospital #2 expressing more concern than those at hospital #1. At hospital #2, management had prohibited the use of all fans on inpatient wards alongside other restrictions while Sydney was subject to public health orders. Participants at hospital #1 were surprised to learn that fans had been banned at hospital #2, and were generally dismissive of any risk.

“Advanced trainee 5: I'm not sure how aerosolizing a hand held fan can necessarily be.

Advanced trainee 2: I don't think there is a significant risk.” (Hospital 1)

The only disadvantage that appeared to dissuade anyone from recommending the fan was a belief that some patients could become overly reliant on the fan, expressed by an allied health professional and nurse in two different focus groups at hospital #2. These clinicians were concerned that patients might become anxious and debilitated if placed in a situation where the fan was not available to them.

“I can find some patients get very over-reliance on the fan as well. They won't move or do anything without having the fan with them. But obviously, yeah, like [participant’s name], I would explore other techniques first before giving them a fan.” Hospital 1, Allied health

Some participants also perceived there to be belief-related barriers among some patients to the fan, including a concern that the fan might worsen a pollen allergy, a cultural belief that drafts could cause colds, and an image issue for some men.

“[Patients say that] ‘when a draft blows through, then I got a cold’ kind of perspective. So, I definitely have had some patients - and sometimes it's from certain cultural backgrounds - that absolutely do not want any wind blowing on their face.” Hospital 1, Advanced trainee

[Unlike women] A lot of men don't carry a bag around, and they're a bit more reluctant to carry the fan with them when they go out.” Hospital 1, Pulmonary rehabilitation

##### Knowledge and beliefs about the fan’s mechanism

Perceptions regarding the fan’s mechanism seemed less important than benefit in determining whether participants recommended the fan, but did determine which sub-groups of patients they chose to offer it. In particular, participants who believed the fan’s mechanism to be primarily psychological recommended it predominantly to patients presenting with comorbid anxiety.

“We also get panic attacks very frequently, like anxious patients. We can recommend [the fan].” Hospital 2, Inpatient nurse

Perceived psychological mechanisms were variously described in terms of a “*placebo effect”* (Hospital 1, Inpatient nurse), “*calming*” (Hospital 2, Inpatient nurse*),* mindfulness (“*just focus on their breathing when they've got the airflow on them*” Hospital 2, Inpatient nurse) and distraction (“*looking at the fan does give them something different to think about*” Hospital 1, Inpatient nurse).

Clinicians in three focus groups also reported reserving the fan for patients in the “*palliative*” or “*end-stage”* (Hospital 2, Inpatient nurses) phase of disease after other interventions had failed.

“I don't bring it up for those individuals who are not quite accepting of their situation, in terms of their diagnosis and how breathless they are, or who lack insight into that. Because then moving on to a strategy to fix that isn't successful. But I wouldn't say it's my last... It's not my last resort. It's in combination with several other non-pharmacological strategies.” Hospital 2, Advanced trainee

However, one participant raised a concern that patients with end-stage disease were sometimes “*too weak*” (*Hospital 2, Inpatient nurse)* to hold the fan, requiring a desktop or pedestal fan instead.

Several participants from the inpatient setting also prioritized other management interventions during an acute exacerbation, considering the fan suitable only for everyday management.

“Yeah. If there's an acute deterioration and their respiratory rate is very high they've dropped their sats [oxygen saturation] I'm not going to be recommending a fan. I'm worried about other things going on, like they're septic or something like that. But, long term … [that’s when the fan might be useful].” Hospital 2, Inpatient nurse

Of participants who thought the fan had a physiological mechanism, the majority had a general understanding that this involved airflow, but only a minority were able to describe this in more detail. Where explanations were offered for how airflow affected the sensation of breathlessness, these included reference to “*pushing air in*” (*Hospital 2, Inpatient nurse*) and “*reducing the work of breathing*” (*Hospital 1, Advanced trainee*), as well as neurological pathways involving various kinds of “*receptors*”.

“So there are lots of inputs that cause dyspnea, and they might be mechanoreceptors, nociceptors, and the like, which feed back centrally to give a perception that someone's not getting enough air. And I suspect, in some patients with chronic lung disease, that those receptors, those mechanisms, might be upregulated. Or they’ve just got barriers or end-stage disease such that those nociceptors are always turned on. And so then other receptors would dampen down those nociceptive pathways, such as a feeling of air, a sensory feeling of air coming across the face. And I think those relieve those sensations by triggering those nerve receptors and those nociceptors to get that feeling that the person's finally getting air.” Hospital 2, Consultant

Only three participants identified involvement of the trigeminal nerve, two of whom were medical staff and one a physiotherapist.

“It stimulates trigeminal nerve and you have inputs through the central respiratory systems that tend to suppress those highest inputs, the sensation of breathlessness and respiratory drive. But yeah, that was my vague understanding…” Hospital 1, Advanced trainee

Several participants likened the fan’s airflow mechanism to fresh air, wind, home oxygen or, in one case, a menthol nasal inhaler, all of which were also perceived to moderate the sensation of breathlessness. One doctor reported advising patients who perceived benefit from home oxygen but did not meet hypoxic criteria to use the fan as an alternative intervention.

“Triggering receptors in the nose and over the face - that'd give you a sensation of air moving across. And that's why I told them [patients] if they're funding their own oxygen even when they don't meet criteria and they've got normal oxygen levels, it's probably the air going over their nose that gives them the relief, not the oxygen itself.” Hospital 2, Consultant

Other participants focused on the cooling effect of the fan either as the sole mechanism or in combination with airflow.

“Usually they [patients] said the cool feeling on their face kind of ‘just helps them breathe better’, in their own quotes.” Hospital 1, Inpatient nurse

In the case of some participants, it wasn’t clear whether they believed airflow and cooling from the fan reduced breathlessness or just made patients more comfortable by compensating for the stuffiness of the inpatient ward or warming effects of equipment.

“So the BiPAPs and the high flows generally blow hot air onto them, or it's humid, so the cool air of the fan just blowing, helps to make them a bit more comfortable while they are on BiPAP or high flow, or whatnot.” Hospital 2, Inpatient nurse

#### Experiential attitude

By comparison, participants’ ‘experiential attitude’ (i.e. how they felt emotionally about recommending the fan) seemed less important than their ‘instrumental attitude’ in determining their fan-related practice. When asked whether they had concerns about the fan appearing too ‘cheap’ or ‘plasticky’, none of the participants agreed this was of concern either for themselves or colleagues (“*If it works, go for it*” *Hospital 1, Advanced trainee*). Indeed, the only emotional disposition expressed towards the fan concerned the empathy that participants felt for patients with chronic breathlessness and commensurate relief at being able to offer them interventions that might be of benefit.

“Having a fan, or knowing that there's one handy somewhere …you can't find anything, and then you tell the patient, "sorry, I can't find anything" - it's a bit frustrating to them. Somehow, you found something, and then you give it [the fan] to them, you can feel their relief for even a little.” Hospital 2, Inpatient nurse

### Normative beliefs

#### Descriptive norms

While patient-reported benefit was the most common reason that participants gave for starting to recommend the fan, a smaller number reported learning about it from other clinicians. Participants reported learning about the fan from both respiratory clinicians (either superiors or other disciplines) and colleagues from specialist palliative care.

“Well I had my bosses suggest it.” Hospital 1, Advanced trainee

“I think the palliative care usually provides some of the handheld fans.” Hospital 1, Inpatient nurse

No participants reported hearing other clinicians criticising the fan or otherwise dissuading others from recommending it and, indeed, expressed incredulity that this would be likely.

“Advanced trainee 2: I don't know why anyone [clinicians] would resist it.

Advanced trainee 3: Yes, it's just a fan.

Advanced trainee 4: It seems, like, such a weird pet peeve to have, like, anti-fan.” Hospital 1

#### Subjective norms

Compared with descriptive norms, participants’ fan-related practice appeared to be more influenced by beliefs and attitudes they held regarding what was expected of them as a clinician. First and foremost, participants perceived their role to centre on patient care, obliging them to support the fan if they believed patients would derive benefits (see ‘Instrumental attitude’ above).

“Anything that will help the patient feel some relief, they [clinicians] won't say anything that they're not happy with it. It's very patient-centred. As long as it’s beneficial for the patient…make them happy…” Hospital 2, Inpatient nurse

However, determining whose role it was to implement the fan faced a lack of clarity at each of the levels of setting, specialty, discipline and clinician. At the setting level, some inpatient clinicians felt that community or outpatient care was better-placed to teach patients how to use the fan at a time when they were not acutely unwell, or at least to reinforce its use if first introduced in the inpatient setting.

“I think it all needs to happen really in the community when they're not in a crisis situation … here [there’s] just not being enough time to actually reinforce it before they're out the door.” Hospital 2, Advanced trainee

“… putting them in place in an inpatient setting then reinforcing them when they follow up in the outpatient setting.” Hospital 1, Inpatient nurse

At a specialty level, some participants deferred to specialist palliative care to recommend the fan and other non-pharmacological interventions, especially those who felt these should be reserved for people with end-stage disease.

“My experience was always thinking previously that it was something that usually palliative care would end up recommending” Hospital 1, Advanced trainee

At the level of discipline, there was an assumption among some nurses and allied health professionals that doctors were more concerned with medical care and thus less likely to consider non-pharmacological management.

“But that's because, I think, doctors are more focused on maybe this blood test or maybe this medication or ‘we need to wean them off oxygen’. Not so much about the long term strategies that people can use when they're not sick.” Hospital 1, Inpatient nurse

However, most doctors reported recommending the fan among other non-pharmacological and pharmacological interventions, and even those who weren’t felt that they should be taking a holistic approach that included this.

Consultant: The patients are coming to us for help; we should give them all the help that we can. So that's why I'm castigating myself slightly for not really having remembered this [i.e. to recommend the fan or other non-pharmacological management strategies]. I don't think that we can put ourselves in little pigeonholes and say, "Oh, that's not my job." I think that historically we could when we had very short waiting times for pulmonary rehab. But even so, we can't count on other clinicians talking about things like that.” Hospital 2

At the individual clinician level, participants generally agreed that any clinician could talk to patients about the fan but, in practice, there was a lack of organisation about who would actually perform this role.

“I think sometimes they can get stuck with “I do this and you do that”. Sometimes, I think in an inpatient setting particularly, everyone assumes someone else has given the education, then no one's given the education. So, I wonder if that's a barrier as well, that some professions think that other professions should be giving that recommendations when it should be all of us?” Hospital 1, Inpatient nurse

### Personal agency

#### Perceived behavioural control

Participants expressed a high level of ‘perceived behavioural control’ or autonomy over recommending the fan. This extended across disciplines, posing less of a barrier to implementation than the normative beliefs outlined above, although this was influenced by ‘environmental factors’, as discussed below.

“You don't have to wait for doctors … you can give it to the patient, and they can use it.” Hospital 1, Inpatient nurse

#### Self-efficacy

In contrast to perceived behavioural control, clinicians’ ‘self-efficacy’ was more variable, especially concerning which type of fan to recommend and how to train patients to use it optimally. Fan characteristics that participants highlighted as important included the batteries not being especially likely to “*fall out*” (*Hospital 1, Inpatient nurse*) or “*run out*” (*Hospital 2, Inpatient nurse*) and the airflow being *“strong enough”* (*Hospital 1, Inpatient nurse*). However, most participants expressed uncertainty about which fan to recommend. One nurse felt that recommending a fan sold and branded by Lung Foundation Australia lent “*credibility*” (*Hospital 1, Inpatient nurse*) that might persuade patients to try it, even though they would also advise the patient that they could get a similar fan cheaper elsewhere.

Participants’ beliefs varied regarding the extent of training and support needed for patients to use the fan optimally, and their confidence in the best approach. Reported approaches varied from minimal (“*I tell them where to buy the fan, tell them where to point it” Hospital 1, Advanced trainee)* to more in-depth explanations about mechanism and situations in which to use the fan, tailoring to each individual patient’s needs. Some participants emphasized the importance of taking time to properly explain the fan and train patients in its use, while others perceived a lack of clinician time to be a key barrier to fan implementation. At one of the hospitals, a doctor reported that a video tutorial was available for patients, but nurses seemed unaware of this.

Participants also varied in the degree to which they combined the fan with other non-pharmacological interventions, and their confidence in doing so. Some participants had never considered using the fan with other interventions, while one allied health professional reported never recommending it alone.

“From a physio perspective, with all our other breathing strategies as well, it works well, positioning, timing, pacing activities. So, it's never on its own … It's an adjunct to what we would normally do.” Hospital 2, Allied health

### Environmental factors

Environmental factors were unique among IBM constructs in impinging on participants’ ‘perceived behavioural control’ to recommend the fan, especially the limited availability of fans in the hospital setting and assumption that costs would be prohibitive in making them widely available. The lack of hand-held fans on the ward meant that participants who worked in this setting relied on using desktop fans. The number of these often failed to meet demand. Also, desktop fans could not be taken home on discharge for portable use during activities of daily living. When asked about resourcing hand-held fans, participants at both hospitals assumed that any funding would be short-lived and run out, with one doctor resorting to buying fans at his own expense. While participants perceived that patients could reasonably expected to buy their own fans when back in the community, limited mobility was sometimes considered a barrier to access.

“These individuals, sometimes … aren't capable of going to the shop themselves to go get it.” Hospital 2, Advanced trainee

### Salience and habit

The poor availability of fans in the inpatient setting was perceived to impede implementation not only due to access but also because it reduced the ‘salience’ of fans and meant that nurses were not in the ‘habit’ of recommending them. Nurses reported care on the wards to be time pressured and often procedurally-driven, with the result that any aspect of care not included in protocols was unlikely to be implemented.

“You get that sort of tunnel vision, not necessarily task-oriented, but it's not part of your protocol. If we knew we had heaps of them there, I think we'd see an increase in people offering them because you've got them to offer.” Hospital 2, Inpatient nurse

In the absence of fans being embedded into ward routine, nurses reported that only patients who brought a fan with them or explicitly requested one tended to receive an opportunity to use one.

## Discussion

This is the first study in any specialty or setting to explore clinicians’ perspectives on barriers and facilitators to implementing the hand-held fan for the relief of chronic breathlessness. The most influential factor determining whether clinicians recommended the fan was a belief that there were benefits for patients, and these outweighed any disadvantages. Understanding of mechanism did not determine whether participants recommended the fan but did determine which patient sub-groups they targeted. In particular, clinicians who believed the mechanism to be largely psychological recommended it predominantly to anxious patients. Some clinicians also reported reserving the fan only for palliative/end-stage patients and/or after other interventions had failed. Other barriers included a belief that patients could become over-dependent on the fan, a lack of clarity about whose role it was to implement it, and what advice to provide patients, as well as limited access to fans in the hospital setting. Few clinicians reported implementing the fan in combination with other interventions. We found few differences in perspective as a function of discipline beyond the views expressed by one or two individuals, limiting broader inference about this as an important factor.

These findings suggest that implementing the fan in specialist respiratory care may require behaviour change strategies targeting clinicians’ ‘capability’, ‘opportunity’ and ‘motivation’ by means of clinician- and service-level interventions [39]. Given relative autonomy in recommending the fan, ‘motivation’ appeared to be the most important factor in driving intention to recommend the fan. Most clinicians in our focus groups were unfamiliar with published evidence for the fan’s effectiveness from RCTs or related guideline recommendations. Most guidelines recommending the fan for chronic breathlessness either focus specifically on cancer [17, 18] or are for palliative care more generally [40–42], with only the New Zealand COPD Guidelines [43] and a ‘workshop report’ from the American Thoracic Society (ATS) targeted at specialist respiratory clinicians [9]. The fan could be recommended across a wide range of respiratory guidelines and clinical pathways, including those for pulmonary rehabilitation and inpatient or outpatient management of breathlessness. Education on the fan could also be included in under-graduate and post-graduate curricula for various disciplines, as well as ongoing training. As highlighted by a participant in the current study, there is no mention in the Royal Australian College of Physicians curriculum for respiratory advanced training [44]; nor is it included in the palliative care curriculum [45]. A recent pre/post study showed that knowledge, beliefs and attitudes towards chronic breathlessness and its management were improved by a 3-day workshop [46]. However, such training would need to be less resource-intensive and implemented at a service-level to reach clinicians most in need rather than ‘preach to the converted’ who are likely to self-select to attend.

Importantly, guideline recommendations and training on the fan should encourage clinicians to offer it alongside disease-directed treatment as ‘first-line’ management of chronic breathlessness, regardless of COPD stage. RCTs evaluating the fan have focused on severity of breathlessness as their inclusion criterion rather than stage of disease [47, 48]. While patients with more severe breathlessness are likely to have more advanced disease, a decision to recommend the fan should similarly be driven by symptom severity and impact rather than disease characteristics. As recommended by the ATS guidelines, the fan can also be used to manage an acute-on-chronic breathlessness episode or ‘crisis’ [9], as well as for everyday remediation. Importantly, the fan can be recommended liberally not only because of the likelihood that patients will benefit but also because of its lack of associated harms.

In addition to behaviour change strategies targeting clinician ‘motivation’, there is also a need for strategies to increase clinician ‘capability’ to implement the fan. Of those participants who recommended the fan, relatively few were knowledgeable about how to implement its use in combination with other interventions. Combined use of interventions is encouraged by a clinical framework called the ‘breathing, thinking and functioning’ (BTF) approach, which acknowledges and addresses the inter-relationships between cognitive and behavioural reactions to chronic breathlessness [49]. Evidence to date suggests the fan’s mechanisms may extend across all three domains by targeting both peripheral and central afferent modulators of breathlessness sensation [28, 50]. Moreover, patients in other studies have reported using the fan to complement breathing techniques and pacing and, in rarer instances, to replace at least some use of beta-agonist metered dose inhalers or home oxygen [8, 16]. These findings suggest that the fan may have potential to influence all three domains of BTF in partnership with other interventions. Further research is needed to refine our understanding of the fan’s contributions and inform education for clinicians and patients on its optimal, integrated use.

Participants’ uncertainty regarding the optimal way to train patients in using the fan is commensurate with the lack of evidence on these questions to date. Training videos available online to date provide guidance to patients on how to use the fan [51–57], but no evaluation has been conducted to confirm optimal content. Like some participants in the current study, authors of one RCT concluded that it is important to explain how and when to use the fan and how it might work, so as to legitimize the fan [58]. This is consistent with evidence from qualitative research that many patients are surprised that such an inexpensive, everyday item can be so effective [8]. However, further research is needed to establish the minimal education that patients require to benefit from the fan to ensure this is feasible within the limited time that clinicians have available, as highlighted by participants in the current study.

Participants were also unsure what type of hand-held fan to recommend to patients, which a recent study has gone some way to answering [59]. In this study, 33 participants with COPD who were attending a pulmonary rehabilitation program trialed 5 different fans in random order. Patient preference was related to perceived strength and pleasantness of airflow, and measured airflow at 30 cm. Further research is needed to test whether these same characteristics are also associated with better breathlessness-related outcomes.

Finally, participants cited availability of fans in the hospital setting as an environmental barrier that decreased the ‘opportunity’ for implementation. Given that fans are effective for increasing physical activity and functioning and may prevent avoidable hospital presentations, making them routinely available to hospital patients seems likely to offer an excellent return on investment. A US cost comparison of interventions for improving exercise tolerance in patients with COPD concluded the fan might cost just $12 per year compared to many thousands of dollars for other interventions in widespread use [60]. Making fans part of routine care might also address the barrier that participants raised regarding lack of clarity about whose role it is to implement the fan.

Reports of fan-related benefit from clinicians participating in the current study are consistent with research showing that patients perceive benefits to breathlessness intensity, physical activity and functioning, and anxiety [3, 4, 7, 8]. Participants’ perceptions of disadvantages were also mostly consistent with previous research, which has similarly suggested that these are limited to an unproven concern regarding transmission of COVID-19 and other airborne infections, and—for a small number of patients—perceptions of stigma [8]. However, participants also perceived two new disadvantages not previously reported in the literature, namely a concern among some cultural groups that air flow itself may cause illness, and a risk that patients could become overly-dependent on the fan, limiting their functioning in situations where the fan might not be available. These concerns were raised by only one and two participants respectively, suggesting that future research is needed to appraise transferability. To date, the only relevant research we are aware of was a recent case study that explored the potential for interventions like oxygen to become “objects of both safety and imprisonment” [61].

### Strengths and limitations

The strengths of this study relate to its in-depth focus on just one non-pharmacological intervention and inclusion of a range of disciplines across 9 focus groups. However, transferability of findings are limited by its focus on specialist respiratory clinicians working predominantly in inpatient services at only two metropolitan hospitals, and junior rather than senior medical staff. Future efforts to understand and address barriers to fan implementation should extend to the outpatient and community respiratory settings, where the fan has potential to support physical activity and confidence for daily living [7, 8]. Given estimates from population-based research that nearly one in ten people aged 15 years or older [62] and a quarter of people aged over 70 [63] in the general community may experience chronic breathlessness, there may also be a case for promoting implementation of the fan alongside other evidence-based non-pharmacological strategies in primary care.

It should also be noted that, while our recruitment strategy was effective in including a range of fan-related practice, there was likely to be at least some sampling bias towards clinicians with more positive knowledge, beliefs and attitudes towards the fan, with the implication that our findings may have under-estimated barriers to fan implementation across the specialty more generally. While the focus group facilitator tried to maintain a magnanimous and open regard to the full range of fan-related perspectives, participants were likely aware of the study’s positive disposition towards the fan so may have been biased towards reporting more favourably to conform and be courteous. We may also have overlooked promising avenues for enquiry that would have been identified by involving consumers in designing the study. Finally, there is potential for use of the IBM during analysis to have limited researchers’ interpretation of the data. To protect against this, analysis began with inductive coding by researchers ‘outside’ the clinical specialty before transitioning to interpretation using the IBM and input from ‘inside’ researchers with nursing and medical perspectives from within specialist respiratory care.

## Conclusion

Service- and clinician-level interventions are needed to implement hand-held fans in specialist respiratory services for chronic breathlessness in patients with COPD. Behaviour change strategies are needed to increase clinician capability, opportunity and motivation to recommend the fan as first-line intervention for breathlessness (rather than reserving it for patients who are anxious or palliative/end-stage) and integrating it with other strategies. Making fans available and incorporated into routine care in hospitals will overcome the main environmental barrier to implementation and is likely to be cost-effective.

## Data Availability

The data that support the findings of this study are available from [third party name] but restrictions apply to the availability of these data, which were used under license for the current study, and so are not publicly available. Data are however available from the authors upon reasonable request and with consideration regarding whether further ethics approval will be required.
